# Prevalence of brucellosis in livestock keepers and domestic ruminants in Baringo County, Kenya

**DOI:** 10.1371/journal.pgph.0000682

**Published:** 2022-08-15

**Authors:** Peter N. Lokamar, Moses A. Kutwah, Elly O. Munde, Dickens Oloo, Harrysone Atieli, Sussy Gumo, James M. Akoko, Collins Ouma

**Affiliations:** 1 Department of Public Health, School of Public Health and Community Development, Maseno University, Kisumu, Kenya; 2 Department of Disease Surveillance and Epidemic Response, Ministry of Health, Nairobi, Kenya; 3 Kenya Nutritionist and Dieticians Institute, Nairobi, Kenya; 4 Department of Clinical Medicine, School of Health Sciences, Kirinyaga University, Kerugoya, Kenya; 5 Department of Zoology, School of Physical and Biological Sciences, Maseno University, Kisumu, Kenya; 6 Department of Theology and Philosophy, School of Arts and Social Sciences, Maseno University, Kisumu, Kenya; 7 Department of Biomedical Sciences and Technology, School of Public Health and Community Development, Maseno University, Kisumu, Kenya; 8 International Livestock Research Institute, Nairobi, Kenya; National Institute of Neurosciences & Hospital, BANGLADESH

## Abstract

Brucellosis is widely distributed in more than 170 countries around the world, where it poses a huge threat to animal husbandry and human health. Brucellosis is a worldwide re-emerging zoonotic disease that poses serious public health problems in many developing countries including Kenya. However, prevalence of brucellosis has not been determined in Baringo County, Kenya, yet there is a continuous movement of cattle resulting from trade and grazing, thus predisposing many herds to brucellosis infection. We investigated the sero-prevalence of brucellosis in humans and domestic ruminants: sheep, goats, cattle and camels among livestock keeping communities in Baringo County, Kenya. In addition, we analyzed the seropositive samples for molecular detection of *Brucella* species. The study adopted a cross-sectional survey using quantitative data collection methods. The diagnosis was carried out using a competitive enzyme-linked immunosorbent assay (c-ELISA) and the real-time PCR assays. The sero-prevalence of brucellosis among human blood samples was 0.6% (n = 4/640) in Baringo County. About 22.30% (n = 143/640) of animal blood samples examined tested positive for *Brucella* genus-specific ELISA test. Cattle had a high prevalence of 22.88% (n = 93/322) followed by camels 20.00% (n = 21/105), goats 15.48% (n = 24/155) and subsequently sheep at 8.62% (n = 5/58). Overall, 7.5% (n = 6/80) of the seropositive samples amplified with the genus-specific primers. *Brucella melitensis* was detected in one out of the six genus positive samples, while none amplified with the *B*. *abortus* target. Even though there was high prevalence of brucellosis among livestock in Baringo County, the highest prevalence was invariably noted in cattle, followed by camels, goats and sheep, respectively. Livestock keepers had low prevalence of brucellosis. This implies that there was low risk of transmission of brucellosis between livestock keepers and their livestock.

## Introduction

Globally, brucellosis is an endemic zoonotic disease that has devastated the livestock industry. According to small-scale livestock owners in most of the developing countries in Asia and Africa, it is the second most frequently reported endemic zoonotic disease to the World Organization for Animal Health (OIE) and regarded as the most devastating trans-boundary animal diseases, which causes significant trade obstructions. Brucellosis is caused by bacteria of the genus *Brucella*. According to previous observations, extensive grazing, large herd sizes and free grazing are some of the risk factors associated with brucellosis. Transmission of the disease can also be due to livestock movement from one geographical region with infection to another as well as hygiene factors [1–3]. Approximately 500,000 humans are infected yearly, however, this infection varies widely in humans and animals with *Brucella abortus*, *Brucella melitensis*, and *B*. *suis;* infecting cattle, small ruminants, and swine, respectively. These three species are of particular importance in human and livestock infections worldwide. The worldwide economic losses because of brucellosis are extensive not only in animal production and reproduction, but also in human health.

In Sub-Saharan Africa, the prevalence of brucellosis in humans varies from 5–55% in different African countries, while in domestic ruminants it ranges between 8–46% [4]. Infection in humans manifests as a range of non-specific clinical signs including general malaise fatigue, arthritis, and fever. Brucellosis can be transmitted to people through inhalation, contact with infected animal fluids and parts such as abortion, fetuses, placental constituents, and vaginal materials. Humans also get infected via consumption of unpasteurized dairy products and under-cooked meat products [5–8]. Animal production systems among pastoralist communities in Africa is extensive but lack implementation of properly disease control and surveillance systems. Animal ownership has for long been recognized as the key risk for exposure to *Brucella spp*. infection through direct contact with infected animal materials and consumption of raw milk and infected meat. In animals, wide range grazing, large herd sizes and uncontrolled grazing are some of the risk factors associated with infection of brucellosis [9–11]. Brucellosis exhibits an array of clinical signs of great economic importance to infected small scale livestock farmers, meat and milk industry and human communities which include poor weight gain, abortions, and other reproductive disorders such as reduced fertility, stillbirths, weak calves at birth, retained placenta and longer calving intervals in female animals and a substantial decline in milk production [12]. The acute and chronic symptoms of the brucellosis can result in a significant loss of man-hours.

In Sub-Saharan Africa, brucellosis is endemic in countries with extensive pastoral production systems, large herd size and free grazing being some of the risk factors associated with brucellosis and where surveillance and control are rarely implemented [13]. Transmission of the disease is also due to livestock movement from one geographical region with infection to another coupled with hygiene factors. Several empirical studies around the globe have related human sero-prevalence to that of animals invariably across husbandry systems [14–16]. Some findings have postulated that husbandry systems might trigger animal sero-positivity while others find no association between these systems and infection [17, 18].

In LMICs, the prevalence of brucellosis in animals and humans is largely unknown owing to several challenges such as weak or non-existent surveillance systems even with variations on the pastoral systems. In resource-limited settings, including Kenya, disease control approaches are typically focused towards diseases with major dramatic effects. Likewise, programs containing approaches of brucellosis intervention are scarce. Low awareness of zoonoses also facilitates brucellosis transmission amongst livestock and humans. In view of this, brucellosis remains endemic, emerging, and reemerging neglected disease that continues to be a major public and animal health threat in developing regions of the world [19].

In Kenya, livestock production is a well-established economic activity for societies that inhabit the high rainfall zones for dairy production. Agro-grounded pastoralism, extensive pastoralism, and commercial beef production are common practices for people in the arid and semi-arid lands (ASAL) [20]. Nevertheless, the reemerging tropical vector-borne and infectious diseases in animals hampers animal production and international livestock markets. Detection of brucellosis remains to be challenging in developing countries like Kenya. Rose Bengal Plate Test (RBPT) is the main frequently used conventional screening test for brucellosis in animals [21, 22]. Human brucellosis has been detected through serological tests particularly for small livestock keeping groups and for patients visiting hospitals with brucellosis like symptoms. The reported prevalence ranged from 0.6% to 35.8% in humans [23, 24]. However, a higher county level prevalence of 46.5% amidst humans was reported in Marsabit County from a study conducted for a set of three counties (including Marsabit) to establish the sero-prevalence and risk factors attributed to brucellosis among humans and their livestock [24].

Two recent studies have investigated brucellosis among camels. The first recorded a prevalence of 11.1% in camels in Marsabit County [24] while the other recorded a prevalence of 0% in five camels tested within West Pokot County [25]. A few other investigations also stretched their surveys on the prevalence of *Brucella* antibodies in milk in Kenya. One used the Milk Ring Test and recorded that 22% of the analyzed 150 pooled milk samples were positive for brucellosis in Kahuro District, Murang’a County [26]. Within the current study carried out in Baringo County, 230 individual farm bulk milks were collected from farmers and analyzed and 24% were positive for brucellosis. Certainly, this prevalence in raw milk, even though not directly associating individual animal prevalence in cattle, postulates brucellosis prevalence among cattle in Kenya and is therefore a risk of transmission to consumers of unpasteurized milk.

In Baringo County, Kenya, cattle, camels, goats, and to some degree sheep are the main livestock animals that are reared by the inhabitants of this region; Tugen and Marakwet pastoralists. The pastoralists in these settings herd these animals together and this practice is documented in studies as one of the alleged risks of transmission of *Brucella* infection. Comprehensive studies on the sero-prevalence of brucellosis among ruminant animal species and occupationally linked humans in Baringo County is scarce yet this is a region of zoonotic significance. It is on this background that this study sought to establish the sero-prevalence and molecular species identification of brucellosis among domestic ruminants and humans in Baringo County pastoral setting with an end goal to develop evidence-based control strategies in Baringo County that will be of public health significance.

## Methods

### Study area

Baringo County is situated in the Rift Valley Region and shares borders with 8 counties namely, West Pokot to the North West, Turkana to the North, Samburu to the North East, Laikipia to the East, Nakuru to the South, Kericho and Uasin Gishu Counties to the South West, and Elgeyo Marakwet to the West. The County is divided into 6 Sub-Counties, namely Baringo South, Mogotio, Eldama Ravine, Baringo Central, Baringo North and Tiaty. It is predominantly inhabited by the Tugen, Pokot and Ilchamus ethnic groups, who are livestock keepers; with minority groups such as Endorois, Nubians, Ogiek, Kikuyu and Turkana. The Tugens mostly practice agro-pastoralism. This mixture of land use allows for complex human animal interactions and interphase usually compounded by the high population density and diversity [12]. It is these complex dynamics that our study was aiming to unravel with respect to brucellosis. Baringo County is classified as arid and semi-arid regions. Most parts of East Pokot, Baringo Central, Baringo South, Baringo North and Mogotio sub-counties are arid and semi-arid except for Eldama Ravine Sub-County, which is a highland zone. The rainfall varies from 1000 mm to 1500 mm in the highlands to 600 mm per annum in the lowlands. The sub-counties due to their varied altitudes receive different levels of rainfall. Eldama Ravine Sub-County receives the highest amount of rainfall. The lowlands sub-counties of Mogotio, East Pokot and Baringo North receive up to 600 mm of rainfall per year. The region is occupied by nomadic communities that qualify it as at higher risk region for the prevalence of brucellosis [27].

### Study design

In a cross-sectional study involving quantitative approach of data collection in Baringo County, 8 locations in Koibatek and Marigat sub-counties were targeted: Torongo, Koibatek, Ravine, Lembus Kwen, Marigat, Eldume, Kimalel and Loboi. The study targeted household heads and domestic ruminants; cattle, goats, sheep and camels. Livestock keepers who were in close contact with the animals were the key respondents for interview and were enrolled as study participants.

### Study population and sampling procedures

#### Study population

The study population consisted of farmers, herders and their livestock (sheep, goats, cattle and camels) from Koibatek and Marigat Sub-counties in Baringo County who were in close contact with their livestock. Majority of livestock were predominantly owned by pastoralists who migrate throughout the dry season looking for water and pastures in small groups of families or in large groups of villagers.

#### Sample size determination

The sample size was determined by Cochran formula (1977) which allowed for the calculation of an ideal sample size given a desired level of precision of 5%, 95% confidence interval and 30% estimated proportion of attribute present in the population. Based on these estimates, 640 animal blood samples (322 cattle, 105 camels, 155 goats and 58 sheep) in 640 households with 640 human blood samples were collected.

#### Sampling procedure

Probability sampling techniques using cluster and simple random sampling methods were used to practically access households that had domestic ruminants. A random sample of 50 villages was selected using a table of random numbers, which gave 30 pastoral villages from Marigat and 20 agro-pastoral villages from Koibatek. The data collection team comprising of laboratorians and veterinary officers who worked in pairs were recruited for purposes of data collection. The team was trained on the research protocol, data collection instruments and ethical issues pertaining to the study. Pretesting of the data collection instruments was done in one of the nearby villages that were excluded from the study. The data collection tools were then customized wherever necessary prior to actual administration. A pair of two enumerators covered at least one village in a day to administer a minimum of 8 questionnaires at random and these were uploaded in Open Data Kit (ODK) in real-time. Once the team was within the prescribed geocode, the compound to be assessed was identified using the ‘spin bottle method.’ Using a flat surface, the enumerator could spin the bottle until it settled and take the direction facing the mouth of the bottle until he/she reached a household with a domestic ruminant.

The first household in that direction was selected and the team administered their questionnaire following informed consent. Once the enumerator finished administration of the questionnaire, he or she stood at the door of the just completed house and spun the bottle again to pick the direction of the mouth of the next bottle. The enumerator again walked to the next household until all eight eligible households were interviewed. An enumerator that reached the end of the village before completing the numbers required, went back to the center of the village, and span the bottle once again. In case the enumerator double selected the previous household, that household was excluded, and the exercise was repeated until another eligible household with domestic ruminants was selected.

The questionnaire captured data on socio-demographics, animal ownership data such as handling, animal product consumption of animal products practices, health-seeking behavior and awareness of brucellosis. For every sampled animal, individual animal level risk aspects were captured; sex, age, method of production, history of abortion, breed, herd size, animal management system, vaccination history, history of introduction of new animals into the herd and presentation of brucellosis like symptoms. This dataset was reported in our previous publication [12]. The assessed households were sensitized on clinical presentation and on signs and symptoms associated with brucellosis and asked to present themselves to nearby health facilities distributed across the study region for medical assistance should they present with the sensitized signs or symptoms associated with brucellosis.

### Sample collection procedure for animals

#### Methods of data collection for animals

Livestock selected for sample collection were individually restrained and 5 ml blood collected in plain vacuum plastic tubes (Vacutainer). Using the halter, the head was elevated slightly, drawn to the side opposite the jugular vein to be sampled and tied to a stationary surface. The vein was occluded by digital pressure in the jugular groove low in the neck. A vacutainer needle, attached to a vacutainer holder, was placed into the distended jugular vein at approximately a 45^0^ angle (angle varies with depth of vein) cranial to the occluding digit. When positioned in the vein, a vacutainer was inserted onto the needle and a blood sample collected. When the desired volume was collected, the occluding pressure was removed. The tube was detached from the needle and the needle removed from the vein. The samples were then labelled and transported in a cool box and ice packs (~4°C) to the hospital laboratory, where they were centrifuged on the same day of collection at 5000 rpm for 5 minutes to obtain serum. The serum and blood samples were transported in freezers and stored at −20°C till testing at Maseno University Laboratory, Kisumu, Kenya.

#### Methods of data collection for humans

Prior to bleeding, the medial site of the elbow region was disinfected using cotton wool soaked in methylated spirit. Blood was aseptically collected from the brachial vein using a disposable 5ml syringe by laboratory personnel. The blood was immediately transferred into a plain vacutainer (Red top) and assigned a unique identification number. After centrifugation, the serum was kept at -20 ^0^C in a freezer till analysis. Approximately 1 ml extracted serum from each human subject was aliquoted into cryotubes for this study. All human sera were also transported at −20°C until further molecular and serological testing at Maseno University Laboratory, Kisumu, Kenya.

#### Other methods of data collection

Quantitative data was collected using the Open Data Kit (ODK) software that captured the demographic characteristics, location signs and symptoms of brucellosis, abortions, treatment, perceived socio-economic effects on livestock production and reproduction performance [12]. These were pre-tested and customized accordingly prior to actual administration. The data collection exercise was conducted in Tugen, the local language, Kiswahili or in special cases, where the respondent was knowledgeable, in English.

#### Questionnaire interview method

A brief structured questionnaire was administered to the head of the household by an enumerator for a period of 35 min and covered specifically animal’s sex, location, history of abortion and retained placenta. For human sampling, information on the age, gender, and location of residence of each sampled human participant was recorded (See S1 Dataset).

#### Blood sample testing rationale

This study’s overall aim was focused on the serological and molecular epidemiology of *Brucella* in Baringo County, Kenya. Therefore, the initial laboratory procedure was to establish the serological c-ELISA and thereafter, perform DNA extraction techniques for all positive samples for PCR testing to detect the genus *Brucella* and to identify *Brucella* species. Results for the initial serological c-ELISA guided the subsequent molecular tests and analysis for *Brucella* species in humans and livestock in Koibatek and Marigat sub-counties of Baringo County, Kenya.

#### Brucella antibody ELISA

The animal *Brucella* antibody tests were performed using the PrioCHECK *Brucella* Antibody 2.0 ELISA (ThermoFisher Scientific, USA), while human Brucella was performed using the Human Brucella-IgM ELISA kit (MyBiosource, USA), as per manufacturer’s instructions. These were indirect ELISA for the detection of antibodies against *B*. *abortus* and *B*. *melitensis* in the serum obtained from humans, cattle, camels, sheep and goats. In brief, the serum samples were dispensed in the coated wells of a microtiter plate. Antibodies directed against *B*. *abortus* and *B*. *melitensis*, present in the test sample, were bound to the antigen during incubation. The bound antibodies were then detected using an anti-Ig monoclonal antibody, conjugated to an enzyme that generated a color signal. The color development occurred when specific antibodies against *B*. *abortus* or *B*. *melitensis* were present in the test sample, which then indicated the presence of *Brucella* antibodies in the sample.

### Molecular analysis and speciation

#### DNA extraction

DNA extraction from the human samples were performed using the DNeasy Blood &Tissue Kit (Cat #s 69504 and 69506) as recommended by the manufacturer (ThermoFisher Scientific, USA). About 20 μl of proteinase K was pipetted into a 2 ml microcentrifuge tube and 100μl anti-coagulated treated blood added with the volume adjusted to 220 μl with PBS. About 200 μl of buffer was added and mixed thoroughly by vortexing and blood samples incubated at 56°C for 10 minutes. Another 200 μl of ethanol was added and mixed thoroughly by vortexing. The mixture was pipetted into a DNeasy mini-spin column placed in a 2ml collection tube and centrifuged at >6000rpm for 1 minute and the flow through and collection tube discarded. The spin column was placed in a new 2ml microcentrifuge tube. The DNA was eluted by adding 200 μl of buffer AE to the center of the spin column membrane and incubated for 1 minute at room temperature. Finally, this was centrifuged for 1 minute at >6000rpm (Quick Start Protocol). The DNA quality and quantity for each of the extracts were assessed using a NanoDrop 2000c Spectrophotometer (ThermoFisher Scientific, USA) before being stored at − 20°C until PCR was done.

#### Real-time PCR

The real-time PCR was done to detect the genus *Brucella* in the extracted DNA samples using primers and probe nucleotide sequences that target the *Bcs31* gene. The PCR procedure was done as previously described [28]. A sample that generated a clear amplification plot and a corresponding threshold value lower than 40 in one or all the duplicate samples was considered as positive for *Brucella*. A further speciation assay was performed on the entire genus for PCR positive samples using the *B*. *abortus* and *B*. *melitensis-*specific oligonucleotide probes and primers. Similarly, only samples that had both clear amplification plot and a Ct value < 40 was considered as positive for any of the two *Brucella* species.

#### Ethical considerations

Ethical approval was obtained from Maseno University Ethical Review Committee (REF: MSU/DRPI/MUERC/00600/18) and permission to conduct research in Baringo County was obtained from National Commission for Science, Technology, and Innovation permit No. NACOSTI/P/18/4661/26645. Introductory letter to the Chief Officer Livestock and Fisheries for Kabarnet was obtained from the office of the Dean, Maseno University. Research authorization was obtained from Sub-County Health Coordinators: Eldama Ravine and Baringo South through Chief Officers of Medical Services and Preventive and Promotive Services for Baringo County. Informed written consent was obtained from study participants and both privacy and confidentiality were guaranteed throughout the study period. The right to participate in the study was voluntary and the participant enjoyed the right to withdraw at any time without penalty. For confidentiality purposes, unique codes were used for both humans and domestic ruminants in the households where samples and data were drawn.

## Results

### Response rate

The final sample size obtained was 640 animal blood samples (322 bovines, 105 camels, 155 goats and 58 sheep) in 640 households with 640 human blood samples collected from the same households.

### Serological analysis based on ELISA

#### Sero-prevalence of brucellosis among humans (livestock keepers) in Baringo County

This study was conducted in Baringo County with 640 human blood samples drawn from livestock keepers who were in close contact with the animals in the investigated households. Four human samples 0.6% (n = 4/640) were found to be positive for *Brucella* genus specific to ELISA test. Generally, the sero-prevalence for brucellosis varied slightly in Koibatek 0.5% (n = 1/208) and Marigat 0.7% (n = 3/432) Sub-Counties (Table 1).

**Table 1:** Sero-prevalence of brucellosis among livestock keepers in Baringo County.

Variable	Category	Examined (N = 640)	Infected	Prevalence (%)	*P*-Value
**Sub County**	Koibatek	208	1	0.5%	0.748
	Marigat	432	3	0.7%	

Note

N = 640

Prevalence is calculated as (infected/examined) *100

#### Sero-prevalence of brucellosis in domestic ruminants (bovine, sheep, goat and camels) in the locations within Baringo County

Similarly, 640 animal blood samples were collected from ruminant animals; cattle, sheep, goats, and camels reared in the same households. Findings demonstrated that 22.30% (n = 143/640) of all the animals examined were positive for *Brucella* genus through e-ELISA test. The sero-prevalence was considerably high in Koibatek 30.77% (n = 64/208) as compared to Marigat 18.29% (n = 79/432) even though majority of the samples were drawn from Marigat Sub-County (*P*≤0.05, Table 2).

**Table 2:** Sero-prevalence of brucellosis in domestic ruminants (bovine, sheep, goat and camels) in the locations within Baringo County.

Variable		Category	Examined (N = 640)	Infected	Prevalence (%)	P-Value
**Sub-County**		Koibatek	208	64	30.77%	**<0.0001** *
		Marigat	432	79	18.29%	
**Sub-County**	**Koibatek**	Torongo	44	7	15.91%	**0.026** *
		Koibatek	72	22	30.56%	
		Lambus Ekwen	52	24	46.15%	
		Ravine	8	3	37.50%	
		Kabiet	32	8	25.00%	
	**Marigat**	Marigat	165	26	15.76%	0.229
		Eldume	105	26	24.76%	
		Kimalel	69	13	18.84%	
		Loboi	93	14	15.05%	

Note

N = 640

Prevalence is calculated as (infected/examined) *100

* Variables which are statistically significant at (*p*≤0.05)

Further analysis was done to establish sero-prevalence among the locations in each of the two sub-counties of interest. In Koibatek Sub-County, the sero-prevalance was significantly high in Lambus Ekwen location 46.15% (n = 24/52), followed by Ravine 37.50% (n = 3/8), Koibatek 30.56% (n = 22/72), Kabiet 25.00% (n = 8/32) and Torongo 15.91% (n = 7/44) locations (*p*≤0.05). In Marigat Sub-County, Eldume location was significantly the leading with 24.76% (n = 26/105), Kimalel 18.84% (n = 13/69), Marigat 15.76% (n = 26/165) animals being infected with *Brucellas* genus. Animals in Loboi location were the least infected 15.05% (n = 14/93) (*P*≤0.05, Table 2).

#### Sero-prevalence of brucellosis in domestic ruminants (cattle, sheep, goat and camels) among Koibatek and Marigat sub-counites in Baringo County

The study also sought to investigate the sero-prevalence of brucellosis among the four types of ruminant animals kept by the inhabitants of Baringo County. Interestingly, cattle was the highly infected at 22.88% (n = 93/322) followed by camels 20.00% (n = 21/105), goats 15.48% (n = 24/155) and sheep at 8.62% (n = 5/58). The proportions testing positive were significantly different across the animals tested (*P*≤0.05, Table 3).

**Table 3:** Sero-prevalence of brucellosis in domestic ruminants (bovine, sheep, goat and camels) among Koibatek and Marigat sub counites in Baringo County.

Variable		Animal Species	Examined (N = 640)	Infected	Prevalence (%)	*P*-Value
**Baringo County**		Bovine	322	93	22.88%	**<0.0001** *
		Sheep	58	5	8.62%	
		Goat	155	24	15.48%	
		Camel	105	21	20.00%	
**Sub-County**	**Koibatek**	Bovine	199	57	28.64%	<0.0001*
		Sheep	2	0	0.00%	
		Goat	7	7	100.00%	
		Camel	0	0	0.00%	
	**Marigat**	Bovine	123	36	29.27%	<0.0001*
		Sheep	56	5	8.93%	
		Goat	148	17	11.49%	
		Camel	105	21	20.00%	

Note

N = 640

Prevalence is calculated as (infected/examined) *100

* Variables which are statistically significant at (*p*≤0.05)

Investigations were further cascaded down to the two Sub-Counties. Worth to note, is that all the goats 100.00% (n = 7/7) examined in Koibatek while 28.64% (n = 57/199) of the bovines tested positive for *Brucella* genus. No *Brucella* antibodies were detected from sheep. Camels were not kept by the inhabitant of Koibatek Sub-County. In Marigat, the sero-prevalence of brucellosis was higher in cattle 29.27% (n = 36/123) followed by camels 20.00% (n = 21/105), goats 11.49% (n = 17/148) and lastly sheep 8.93% (n = 5/56). The proportions of the animals testing positive vs. negative were statistically significant in the different sub-counties (*P*≤0.05, Table 3).

#### Molecular analysis for *Brucella* species

The results from the serological analysis examined 640 blood samples from the 640 households that participated in the study. However, molecular analysis based on the real-time PCR technique identified 80 species of *Brucella*. In total, 7.5% (n = 6/80) samples were found to be positive for the genus *Brucella*. However, only one out of the six *Brucella* genus positive samples amplified with the *B*. *melitensis* specific target, while none was positive for the *B*. *abortus* target (Table 4). The proportion of *Brucella* genus positive samples was higher in Koibatek 8% (n = 4/50) compared to Marigat 6.7% (n = 2/30). However, this variation between the two regions was not statistically significant (*P* = 0.70).

**Table 4:** Results of molecular analysis using real-time PCR.

Area	Number of samples	Genus positive	*B*. *abortus* positives	*B*. *melitensis* positives	*p-value*
**Marigat**	30	2 (6.7%)	0	0	0.70
**Koibatek**	50	4 (8%)	0	1	
**Total**	80	6 (7.5%)	0	1	

## Discussion

Brucellosis is a neglected bacterial illness that poses a significant risk of infection in humans and animals. It has a substantial economic impact on agriculture as well as public health issues, particularly in underdeveloped nations like Kenya [29]. Humans contract brucellosis by coming into contact with infected animals or eating contaminated raw animal products. In humans, brucellosis causes non-specific symptoms that are likely to pose long-term consequences. Our findings on serological analysis depicts a sero-prevalence of human brucellosis in Baringo County as 0.63%, (n = 4/640), which was lower than the 5.7% and 32% estimated previously in Kiambu and Kajiado Counties, respectively [29]. In addition, the sero-prevalence of brucellosis among domestic ruminants in Baringo County’s pastoral districts was investigated. Overall, 22.30% (n = 143/640) of all the animals examined were infected with *Brucella spp*. These observations were consistent with the previous 7.6% sero-prevalence of brucellosis reported using the indirect ELISA (c-ELISA) in commercial dairy cattle in Chittagong District [30] and the 8.5% sero-prevalence tested using the rapid *Brucella* antibody test kit in the Sirajgonj District of Bangladesh [31]. Recent studies in Kenya show that nomadic pastoralists may not adhere to practices that would reduce *Brucella* infection albeit having considerate knowledge on risk and transmission of the disease, occupational hazards, and cultural practices of consumption of animal products [29].

In this study, the individual overall sero-prevalence with ELISA was 22.30% (n = 143/640). These values are higher than those obtained from previous studies carried out in Mali [32] (4.1%). However, our results were slightly lower than the highest sero-prevalence observed in the country (37.1%) in Marsabit County [24]. The differences in the sero-prevalence’s in the above locations versus that observed in our study could be due to differences in the types of production systems, possibility of cross-transmission of *Brucella spp*. from one livestock host to the other, which occur in different settings. In addition, the high prevalence noted in Marsabit County could be attributed to congregation of animals around communal watering points, keeping of mixed herds, and sharing of grazing sites that have been previously reported to increase chances of brucellosis transmission leading to the high prevalence in such regions [33].

We were also interested in establishing the sero-prevalence of brucellosis in cattle, sheep, goat and camels in the two sub-counties of Baringo County. Interestingly, the prevalence of brucellosis was higher in Koibatek Sub-County at 30.77% (n = 64/208) where most residents engage in mixed farming 43.8% (n = 91/208) than in Marigat Sub-County 18.29%, (n = 79/432) where most of the residents are purely nomadic pastoralists 49.5% (n = 214/432). This observation is in discordant with the results of other studies in livestock where the prevalence was 15.2% in pastoral production system and 4.1% in agro-pastoral areas of the study regions in Ethiopia [34]. Such phenomena observed in our study site could potentially be promoted by the different types of breeding systems used for reproduction in the two regions of Baringo County. About 72.1% (n = 150/208) of livestock keepers in Koibatek Sub-County practice artificial insemination while 95.6% (413/432) of those in Marigat Sub-County utilize natural breeding system for reproduction. However, our study results concur with that of Segwagwe and others [35] in Rwanda which indicated more positive cases among cross-breeds (22.7%) than local breeds (13.8%). Further interrogation on the importance of breeding types and transmission patterns of *Brucella* needs further investigations in such set-ups.

Our analysis currently provide the initial evidence on the sero-prevalence of brucellosis among the four species of livestock kept by the residents of Baringo County: Cattle 22.88% (n = 93/322) followed by camels 20.00% (n = 21/105), goats 15.48% (n = 24/155) and lastly sheep 8.62% (n = 5/58). This is quite important in the sense that it creates a critical understanding of the little known prevalence of brucellosis among domestic ruminants in Baringo County, a region marred with huge practices of nomadic pastoralism in which communities move from place to place in search of water and pasture. It would be prudent to hypothesize that in such settings, sharing of community grazing fields and water points among livestock exacerbates and promotes cross-species transmission of brucellosis [12].

In Koibatek Sub-County the sero-prevalence of brucellosis was highest in goats 100% (n = 7/7), followed by 28.64% (n = 57/199) in cattle. There were no camels assessed while sheep had a prevalence of 0% in Koibatek. In Marigat Sub-County 29.27% (36/123) of cattle, 20.00% (n = 21/105) camels, 11.49% (n = 17/148) goats and 8.93% (n = 5/56) sheep tested positive for *Brucella* antibodies. The results obtained in this investigation confirm the endemic nature of overall brucellosis in domestic ruminants in Koibatek 30.77% (64/208) and Marigat 18.29% (n = 79/432) in Baringo County. These prevalences might not be high given that the herd prevalence is below the cut-off point of 35% while the individual prevalence is more than the cut-off point of 10%. These observations from our sites to some extent, were higher than those observed in the previous investigations in which it was demonstrated that brucellosis prevalence was about 10% in domestic ruminants in Rwanda using the ELISA [36]. The Tugens who reside in Koibatek mostly practice agro-pastoralism and keep small ruminants and cattle, whereas Pokot and Ilchamus who reside in Marigat practice nomadic pastoralism and keep camels, cattle and small ruminants. Pastoralists have a nomadic way of life whereas agro-pastoralists are either transhumant or settled. The environmental conditions of these zones, as well as the farming methods used, may have contributed to the difference observed between our current study and that in Rwanda. Indeed, according to other investigators [32], hot and humid environments favor a rise in brucellosis sero-prevalence. Our current study site is both hot and humid, conditions that favor high sero-prevalences.

The sero-prevalence of brucellosis in cattle was high in both Koibatek 28.64% (n = 57/199) and Marigat 29.27% (n = 36/123) regions. Cattle 50.31% (n = 322/640) formed the highest proportion of domestic ruminants reared by livestock keepers in Baringo County. These findings were lower in comparison to studies carried out in cattle in Adamawa 36.6% [37], and 34.0% in Yobe [38]. However, the outcome of current investigation is higher than 3.5% reported in Ethiopia [11] and 2.77% in Eritrea [39]. These variations could be attributed to the differences in sample size used and agro-ecology, coupled with sharing of grazing fields and watering points (mentioned earlier). Our study also confirmed that bovines are the most preferred livestock species reared in both Koibatek and Marigat regions of Baringo County. This explains why they are the most affected as they are the most abundant to be affected by *Brucella* in the region.

A further novel finding is that all goats 100% (n = 7/7) cross-examined in Koibatek were positive for *Brucella* species as compared to Marigat 11.49% (n = 17/148). This suggests that goats may be over-affected in one region, although this could also be due to the lower sample size in Koibatek than in Marigat Sub-County. This observation requires further investigation especially in the context of more sample sizes in both regions of Baringo County.

Other findings revealed that camels were predominantly found in Marigat with a sero-prevalence positivity rate of 20% (n = 21/105). Camels were only sampled in Marigat and not in Koibatek Sub-County. This could be due to the climatic and ecological niche that favors rearing of camels in Marigat region as opposed to Koibatek region. Even though we had no data to compare across the 2 sub-counties with regards to camels’ sero-prevalences, we highly suspect that the recorded sero-prevalence of brucellosis among camels in Marigat Sub-County was high and could closely also be related to the frequent migration of camels and other herds, which facilitates the sharing of grazing sites and drinking stations, as well as direct interactions between herds, thus increasing their exposure to *Brucella* [40].

The current study showed a lower prevalence of human brucellosis (approximately 0.75%) in Koibatek and Marigat sub-counties relative to other previous studies in Kenya [41]. This could be attributed to relatively lower exposure to human populations in the sampled regions. However, further investigations considering a model in which the geographical location, different production and reproduction systems, time, place and risk factors predisposing livestock and livestock keepers to brucellosis, needs to be performed to delineate the true exposure rates in Koibatek and Marigat sub-counties of Baringo County, Kenya.

The molecular analysis in this study region revealed some key findings: All serum samples from humans tested negative for *Brucella* species. Among the domestic animals (goats, sheep, camels and bovine) reared in Baringo County, only the bovine species tested positive for *Brucella*. Real-time PCR identified *B*. *melitensis* specific target in only one bovine that was reared in Koibatek Sub-County. About 98.8% (n-79/80) did not amplify with either *B*. *abortus* or *B*. *melitensis*-specific primer target. Findings from previous molecular and serological studies in Narok, Marsabit and Kajiado have shown that *B*. *melitensis* is a common species in pastoral communities such Marigat in Baringo County [23].

The distribution of B. *melitensis* was only identified in Koibatek Sub-County as opposed to Marigat Sub-County. This could be attributed to differences in production systems practiced in the two regions of study; in Koibatek Sub-County, the semi-zero grazing production system is at 83.2% (n = 183) and 16.8% (n = 37) in Marigat Sub-County. In other studies [33], semi-zero grazing production system is characterized by animals sharing pasture, watering sources between several herds and/or uncontrolled movement of livestock within the grazing fields thus contributing immensely to exposure to infections pathogens such as *Brucella* among livestock species. Our findings are therefore consistent with similar studies conducted in different regions in Sub–Saharan Africa [24]. Brucellosis is a zoonotic disease and still remains of public health concern given the steady rise in bovine populations in Kenya primarily for meat and other animal products [42].

## Conclusion

Brucellosis is an endemic zoonotic disease in low- middle- and high-income countries that causes devastating losses to the livestock industry including small-scale livestock holders and nomadic pastoral communities. Our study provides initial evidence of the high sero-prevalence of brucellosis in domestic ruminants (camels, cattle, goats and sheep) and humans in Koibatek and Marigat regions of Baringo County, Kenya with *B*. *melitensis* species being picked up with the molecular methods. Close contact between domestic ruminants and human beings is still a critical mode of transmission that should further be explored among pastoral communities in Kenya and other endemic regions. This is indeed true since brucellosis is a threat to human healthcare systems and inhibits the economic potential of people, communities, and nations in areas where economic development is critical to reducing poverty. This together with future findings should guide in developing public health policies aimed at reducing the socio-economic effects of brucellosis in humans and animal populations in Baringo County. Moreover, the national veterinary service must be strengthened to carry out the strategy, which includes increased collaboration between public health, veterinary services under One Health approach. Future studies could fruitfully explore this issue by assessing the impact of brucellosis on the livestock economy, livestock and human health.

Part of the limitation of the current study is that all brucellosis cases were diagnosed serologically by ELISA rather than by culture limiting analysis at the *Brucella spp*. level. However, the species-specific PCR method could have eliminated biasness in our approaches. Our selection of behaviors to include in the exposure scales may not have been sufficiently comprehensive or may have been too exclusive.

This study provided evidence of the presence of *B*. *melitensis* in bovine in Baringo County Kenya, by PCR as opposed to all other livestock species and humans examined in the study. Future studies should consider expanding the range of real-time PCR options to shed more light on all the *Brucella* species in Baringo County. Our findings also confirmed that *B*. *melitensis* has a significant positive association with bovine reared under semi-grazing production system in Koibatek Sub-County.

## Recommendations

The One Health Approach to zoonotic diseases indicates that collaboration of veterinary, medical, public health, economic and social cultural experts are needed to effect a change in disease burden. In addition, animal movement restrictions, and immunization strategies are effective in controlling transmission of brucellosis. There is therefore, need to institute a national control and mitigation strategy in Baringo County and other brucellosis endemic regions to improve food security, household income, and human and animal health in low resource communities. Similarly, resources should be allocated towards research and development of improved *Brucella* vaccines and diagnostic tools as important facets of addressing the problem. Routine surveillance of human brucellosis is also essential to monitor and evaluate the successes of counter measures. High-risk occupational groups must be made aware of zoonotic diseases, educated on precautionary measures to protect themselves and reduce the risk of infection between and among consumers as well as livestock keepers in Baringo County, Kenya.

## Supporting information

S1 Dataset (XLSX)
